# Assessing the Current Limitations of Large Language Models in Advancing Health Care Education

**DOI:** 10.2196/51319

**Published:** 2025-01-16

**Authors:** JaeYong Kim, Bathri Narayan Vajravelu

**Affiliations:** 1 School of Pharmacy, Massachusetts College of Pharmacy and Health Sciences, Boston, MA, United States; 2 Department of Physician Assistant Studies, Massachusetts College of Pharmacy and Health Sciences, 179 Longwood Avenue, Boston, MA, 02115, United States, 1 6177322961

**Keywords:** large language model, generative pretrained transformer, health care education, health care delivery, artificial intelligence, LLM, ChatGPT, AI

## Abstract

The integration of large language models (LLMs), as seen with the generative pretrained transformers series, into health care education and clinical management represents a transformative potential. The practical use of current LLMs in health care sparks great anticipation for new avenues, yet its embracement also elicits considerable concerns that necessitate careful deliberation. This study aims to evaluate the application of state-of-the-art LLMs in health care education, highlighting the following shortcomings as areas requiring significant and urgent improvements: (1) threats to academic integrity, (2) dissemination of misinformation and risks of automation bias, (3) challenges with information completeness and consistency, (4) inequity of access, (5) risks of algorithmic bias, (6) exhibition of moral instability, (7) technological limitations in plugin tools, and (8) lack of regulatory oversight in addressing legal and ethical challenges. Future research should focus on strategically addressing the persistent challenges of LLMs highlighted in this paper, opening the door for effective measures that can improve their application in health care education.

## Introduction

Artificial intelligence (AI) as a field of computer science research aims to maximize the development of software tools that capacitate machine-based simulation of human intelligence within defined parameters [1]. Often described as the pinnacle of information technology of this century, its integration into the greater boundaries of human infrastructure is expected to both fundamentally and permanently revolutionize the ongoing information age.

Large language models (LLMs) represent deep learning architectures called transformer networks [2]. It relies on neural networks that discerns the relationships within and between sequential data. Generative AI models, such as the generative pretrained transformers (GPT), collectively operate under such deep learning neural networks, thereby allowing for the training, processing, and analysis of large quantity of complex data to be possible at an exceptional rate and accuracy [3].

With the power of NVIDIA’s graphics processing units, OpenAI announced a 175 billion parameter language model (GPT-3.0) to the public in June of 2020 [4]. GPT-3.0 has quickly garnered international attention for its ability to summarize, translate, classify, and engage in real time, generating detailed and human-like response to user queries. Since its release, a plethora of domain specific LLMs (such as Med-PaLM) and alternative models (such as Gemini and Claude) with advancements in multimodal capabilities have emerged [5], significantly enhancing the breadth of publicly accessible LLMs.

In May 2024, OpenAI introduced ChatGPT 4o (omni), an advanced iteration built on the GPT 4 model, which continues to establish new standards for LLMs. It incorporated various advancements over its predecessors and contemporary models, including improved performance in complexity, specialization, multilingual capabilities, and resource optimization [6]. Furthermore, OpenAI’s integration of multimodal tools within the GPT interface has enabled the model to comprehend and generate responses based on both visual and verbal inputs. Specifically, the integration of DALL-E, a text-to-image model [7], along with the advanced data analysis feature [8], has expanded the traditionally text-based nature of LLMs into a more versatile, information integrating modality.

Despite the ongoing “AI race” that continues to raise the bar for generative AI technology, its integration into health care education poses significant and multifaceted challenges. In recent years, the shift toward embracing AI in education has gained more momentum, as highlighted by New York City’s decision to rescind its previous ban on ChatGPT [9]. To this end, the primary objective of this review is to identify potential risks and propose effective mitigation strategies that developers of LLMs should adopt to facilitate their successful integration into health care education.

## Risks of Large Language Models in Health Care Education

### Overview

**Figure 1.**
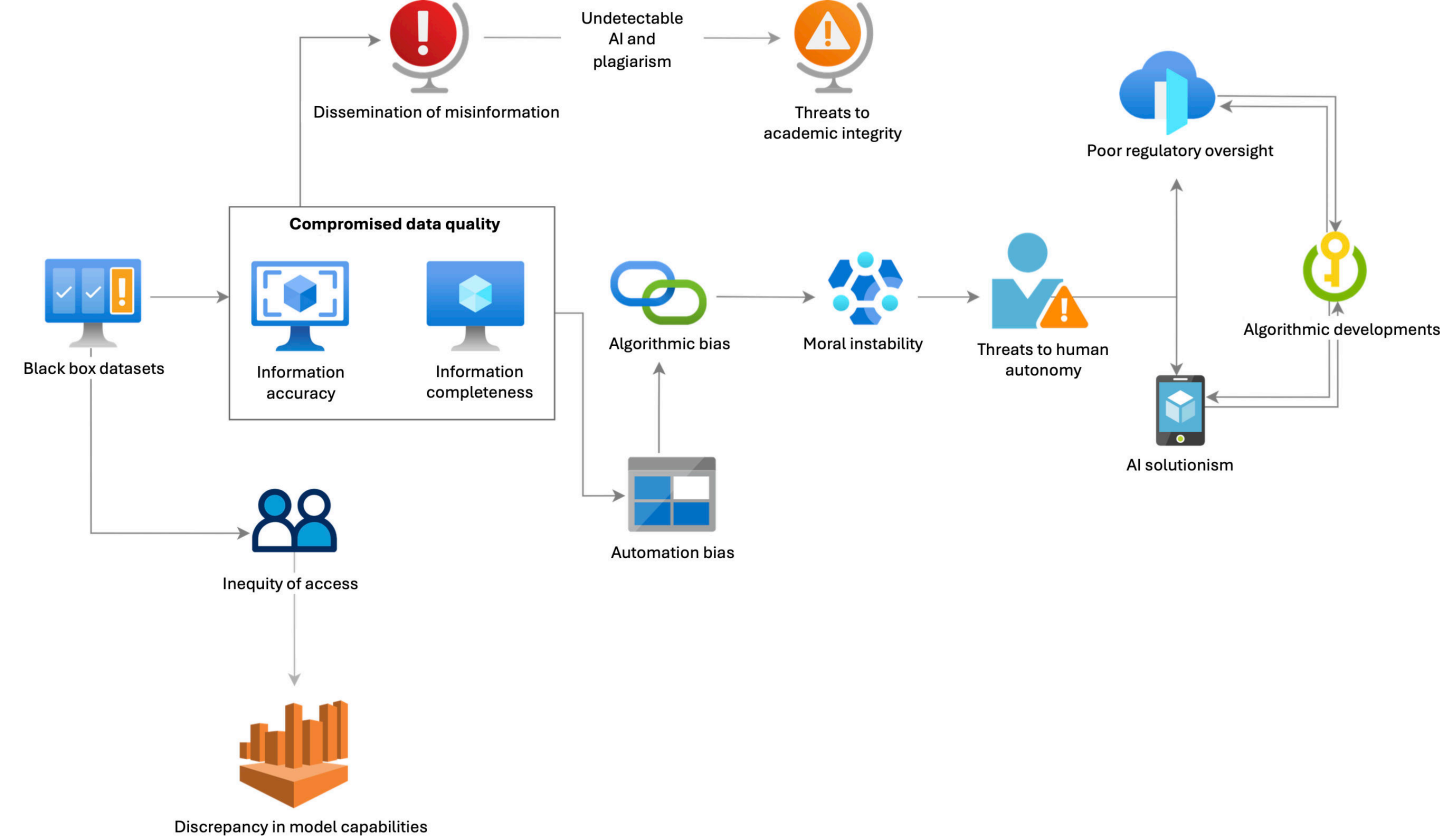
Assessing risks and limitations of large language models in health care education. AI: artificial intelligence.

This section evaluates the current risks and limitations associated with the use of LLMs in health care education. It aims to highlight specific areas of concern that must be addressed at both individual and systemic levels. Figure 1 provides a summary of these issues through a flowchart.

### Threats to Academic Integrity

LLMs, like GPT-4o, provide individualized learning assistance tailored to the specific user demands. The increasing power of LLM’s ability to process nuanced language understanding, along with enhanced problem thinking skills make it incredibly enticing for students to exploit its use under academic settings, both knowingly and unknowingly [10]. With increasing reliance on virtual learning platforms and learning management systems in health care education [11], AI-generated texts in particular compromise academic integrity by blurring the distinction between human and machine ingenuity.

Despite the actuation of detection software tools designed to combat AI-generated plagiarism (such as GPTZero), technological limitations and high error rates of these AI content detectors have been well-demonstrated [12-15]. In July 2023, OpenAI officially terminated its own AI detection software, AI Classifier, citing high false positives and inconsistent performance [16]. Comparative studies further affirm that neither human evaluators nor AI detectors are effective in identifying AI-generated medical literature [13]. Furthermore, generative texts can be easily modified to evade AI detection through simple grammatical adjustments, such as with the addition of adverbs or the use of synonyms [17,18]. Unsurprisingly, the widespread availability of AI-based plagiarism removal tools, such as Writehuman, leverages these strategies to convert AI-generated texts to appear more human-like, which may inadvertently discourage personal authenticity. Therefore, LLM’s flexibility and immediacy in generating original, user-specific contents render the detection of AI-based plagiarism increasingly impractical [17].

The growing inability to differentiate between human and machine-generated writing poses significant threats to the principles of academic integrity, namely “honesty, trust, fairness, respect, and responsibility” [19]. For instance, the ethical controversy surrounding Elsevier’s AI author policy, which led to a publication featuring an AI-generated introductory sentence, underscores the already pervasive exploitation of LLMs in academic writing and research [20]. The long-term repercussions of incorporating LLMs in education, such as the risk of over-reliance, erosion of critical thinking and problem-solving abilities, the deterioration of writing and summarization abilities, challenges associated with verifying information, and many more, have been well discussed [21,22]. In an era of infobesity, health care educators and LLM developers must establish clear policies and expectations that promote transparency and foster dialogue about the use of AI-integrated technology, while actively working to minimize the associated risks.

### Dissemination of Misinformation and Risks for Automation Bias

LLMs are engineered to generate a response by predicting the most probable response from input strings of its users. However, the details of both the data sources and the quality of data, along with specific parameters that are used to train LLMs, continue to remain undisclosed [23-25]. Such “black box” nature of LLMs pose challenges in assessing their reliability and robustness in health care applications [26-28], where information accuracy in health care knowledge is directly associated with the effectiveness of patient outcomes [29]. Any inaccuracies or misinformation generated by these models can manifest into inappropriate treatment strategies, misdiagnoses and professional incompetence that collectively lower the quality of health care delivery [30].

Despite receiving reinforcement learning from repetitive user feedback [31] and significant algorithmic improvements made to address AI hallucinations [32], both ChatGPT-Free and ChatGPT-Plus continue to remain susceptible to disseminating misinformation [24,33-35] and knowledge fabrication [36]. In a comprehensive meta-analysis assessing the performance of ChatGPT-3.5 in medical inquiries, the overall accuracy was found to be 56% [37]. Similarly, in a cross-sectional study comparing ChatGPT-3.5 and ChatGPT-4o with 284 physician-developed medical queries, only 50% of the responses were accurate [28]. Mounting evidence from domain-specific research [25,28,38-42] increasingly highlight significant concerns surrounding the accuracy and reliability of model data, pointing to their consistently poor performance in the context of health care education and clinical decision making.

Furthermore, LLM generated contents are highly vulnerable to committing source-based plagiarism, as it readily produces fabricated or inaccurate reference when asked to provide one [23,32,43]. This makes it impossible for users to reliably track and retrieve source information for verification [44] . Another persistent issue in the programmed nature of ChatGPT is its tendency to present information using bullet points. Although this format appears effective, it can inadvertently spread logical fallacies and misinformation. The hierarchical structure can misrepresent the significance of information, leading to inconsistencies that could unintentionally violate clinical practice guidelines [39].

To mitigate such concerns, OpenAI integrated third-party web plugins for GPT-4o, enabling the model to access the internet in real-time [45]. However, such strategy aggravates automation bias [27], where overreliance on AI capabilities increase the risks for AI solutionism. The dangers of AI solutionism have been highlighted by instances where AI-generated medical literature has successfully deceived human experts in broader, nonspecialized subject fields [14]. As a result, both health care students and clinicians may struggle to fulfill their responsibility to critically evaluate authentic knowledge from unverified information, impeding information seeking and processing abilities [10].

Without stringent regulatory frameworks and transparent disclosure of its training datasets, LLMs will struggle to gain the credibility necessary for integration into health care education and clinical practice. For this purpose, the development of a specialized, scaled-down and health care optimized LLMs, such as Google’s Med-PaLM 2, will be essential. Mitigation steps toward enhancing the reliability LLMs in health care education include prioritizing transparency in training datasets, improving information accuracy within smaller datasets, ensuring the deidentification of medical data, and making algorithmic advancements to minimize AI hallucinations.

### Challenges With Information Completeness and Consistency

Information completeness measures the extent to which a dataset is comprehensive. Data are considered complete when they encompass all required and relevant data fields, without any omissions [46]. LLMs, despite being built on vast body of knowledge, are susceptible to generating incomplete or partial representations of their knowledge dataset. Consequently, this leads to inconsistencies in the quality and comprehensiveness of their outputs [42].

In a study using a 3-point Likert-scale to measure completeness of ChatGPT’s response to 180 medical queries, GPT-3.5 only scored 53.3% in terms of answer comprehensiveness [28]. Similarly, when GPT-4o’s responses to clinical case questions were evaluated using the same scale, the model scored 53.3% in answer completeness [47]. In a different study, majority of the correct medical responses generated by GPT-3.5 were labeled as incomplete, where the omission of decision-making cut-offs, treatment strategies, and durations [38] greatly undermined information completeness. These findings were corroborated in another study, exhibited by GPT-3.5’s lack of insights into treatment efficacy and age-or patient-specific recommendations, resulting in a 45% score in comprehensive output [48]. It is important to note that, when queried individually about such specific components, ChatGPT demonstrates understanding of relevant body of knowledge but fails to perform systemic and comprehensive integration of them under complex user requests.

As a natural extension of these observations, LLMs, such as the ChatGPT series, exhibit limited proficiency in generating outputs based on complex and information-rich inputs. This inverse relationship between input quantity and output quality has been well demonstrated by ChatGPT’s tendency to produce ambiguous response when addressing lengthy medical queries that involve complex clinical context and nuance [49,50]. Furthermore, the GPT series were found to lack human-like understanding required for medical training, often leading to absurd responses [34]. The model’s inability to fully grasp and accurately synthesize complex medical information undermines their reliability, leading to a phenomenon called “model overconfidence” [44] that generate imprecise and erroneous outputs. This supports the narrative that LLMs require robust oversight to fine-tune data consistency for the model to perform reliably [19].

Along with information accuracy, information completeness is a critical component of the overall quality of information generated by LLMs. Developers of LLMs must refine the model’s ability to consistently produce comprehensive and contextually accurate outputs based on improving both the quality and quantity of data parameters. Addressing these deficiencies is imperative in augmenting the applicability of LLMs in health care education.

### Inequity of Access

**Figure 2.**
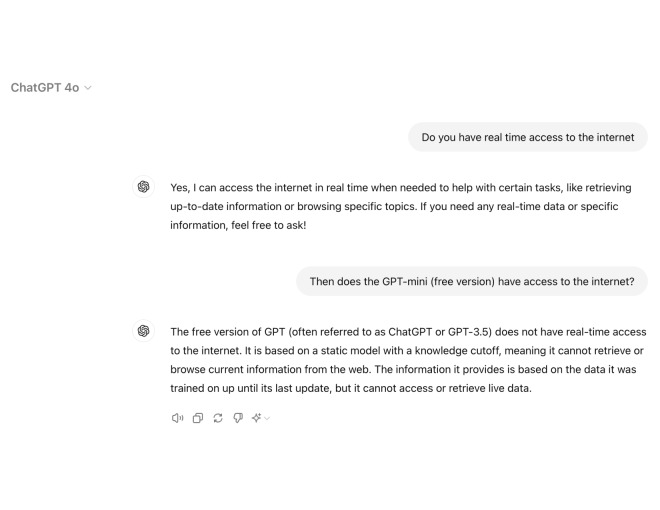
ChatGPT model differences in internet access capabilities.

**Figure 3.**
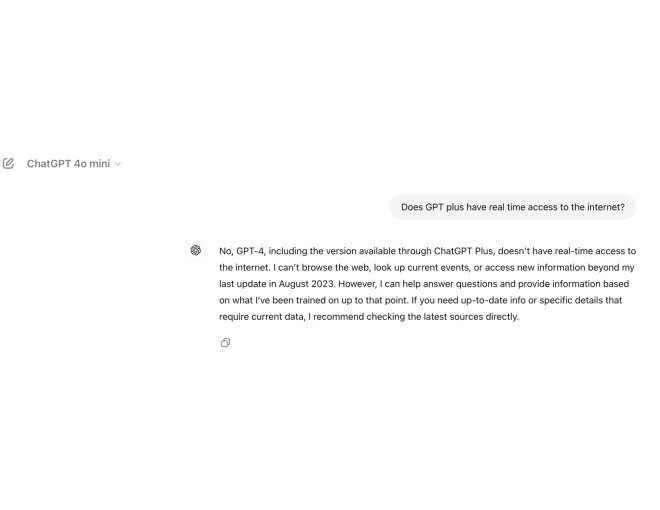
Understanding misinformation risks in ChatGPT-Free.

Monetization of certain LLMs, such as seen by Open AI’s distribution of ChatGPT-Plus by a monthly subscription service [51], raise ethical concerns with regards to fair and equal accessibility to information. The vast majority of LLMs, with the exceptions of Gemini [52] and ChatGPT-Plus (Figure 2), lack real-time web access and, therefore, are incapable of retrieving information beyond their trained datasets. This undermines both the quality and consistency of the information that LLMs present, which renders the knowledge databases of most accessible LLMs presently outdated [9]. Figure 3 demonstrates how misinformation proliferates from outdated databases.

Generative AI tools, like most other automated technological infrastructures, have inherent disparate impacts and thereby are less accessible to those whose training data are unavailable or inadequate for a specific language other than English (disparities in both language [53] and cultural proficiency [54]), those without necessary technology for access and limited digital literacy [55] those with physical disability and or impairment [56], and those with intellectual disability and or psychiatric impairments [57]. The monetary approach to the production of a premium service of GPT introduces economic disparity to the above list, where those that cannot afford subscription fees (US $20/month for ChatGPT-Plus [51]) are at a risk of aggravating digital divide disparity [58].

By this notion, standard search engines greatly outcompete LLM’s use when it comes to both data reliability, uniformity, and completeness [26]. In other words, LLMs that provide incomplete or superficial information, often with significant delays in information currency [27], offer limited value in the fast-paced realms of health care education, research, and clinical practice.

A review (n=21) was conducted to compare information accuracy between the premium and free versions of ChatGPT using studies from PubMed and Google Scholar (Tables 1 and 2). Inclusion criteria were based on analysis focused on health care education and clinical management, categorizing literature by subject matter to calculate mean accuracy differences. Categories were carefully defined to minimize selection bias, distinguishing between subjects such as “question banks” and “board exams,” acknowledging GPT-Plus’s expansive data access compared with that of GPT-Free.

**Table 1.** Comparative analysis of accuracy discrepancies: GPT-3.5 versus GPT-4.0.

Subject and category of comparison	Study	Reported percentage difference in accuracy levels between GPT-4.0 versus GPT-3.5 in corresponding literature, %	Accuracy levels (%) by category (n=7), mean (SD)
Board, entrance, and licensing exams	[59-65]	29.1, 27, 20, 22.2, 29.6, 29, 21.3	25.46 (4.14)
Question banks, mock exams, self-assessments	[66-72]	17.7, 10, 22, 23.8, 20.2, 16.5, 18.7	18.41 (4.47)
Clinical case, clinical questions, referencing	[73-79]	12.3, 27.8, 27, 10, 10.42, 13.6, 8.6	15.67 (8.17)

**Table 2.** Cross-tabulation of performance accuracy of GPT versions.^a^

	Studies reporting an accuracy of ≥70% (+), n	Studies reporting an accuracy of <70% (–), n	Total, n
ChatGPT-4.0 (PLUS)	18	3	21
ChatGPT-3.5 (FREE)	2	19	21
Total	20	22	42

a *P*<0.001. *P* was calculated as follows: *P* = ([a+b]![c+d]![a+c]![b+d]!)/a!b!c!d!n!

This review demonstrates that ChatGPT-Plus significantly outperforms the free version in information accuracy, with statistical significance confirmed by Fischer exact test (*P*<0.001) at a 95% CI. The study sets a 70% accuracy threshold for binary classification, highlighting a profound performance capability between the 2 versions. These findings raise significant concern about the potential for a digital divide, driven by the differential AI capabilities between the paid and free versions of LLMs.

Without a large-scale regulatory measure that dictate the quantifiable discrepancies in model capabilities between its free and premium editions, the ongoing rapid evolvement of AI technology will continue to create a stark polarity in equitable resource distribution [80]. In other words, development may further aggravate discrepancies in knowledge access and information availability in resource-constrained environments, particularly jeopardizing information accessibility in low-income demographics or users of 3rd world countries [55].

### Risks of Algorithmic Bias

LLMs propagate algorithmic biases rooted in their training data, often leading to medically inaccurate and discriminatory responses. While no algorithm is designed to be innately and or deliberately discriminatory [81], they inevitably inherit and amplify existing sociocultural and historical biases embedded in their training [82,83]. In health care education and clinical practice, where racial misinformation persists [84], the integration of LLMs risks perpetuating biases, potentially leading to clinical errors and malpractice with significant repercussions.

LLMs, such as the ChatGPT series, Gemini, and Claude, have been demonstrated to perpetuate race-based biases in medicine, which unfortunately reflect and exacerbate existing health care disparities [85]. These models often recommend inconsistent treatment strategies for patients of different racial backgrounds, rooted in flawed assumptions about biological variations, such as differences in pain tolerance [86] and kidney function [87]. Furthermore, the GPT series has been criticized for promoting demographic stereotypes, which disproportionately associate certain diseases with specific races, ethnicities, and genders [88].

Furthermore, research has highlighted the influence of racial classification on the outputs generated by ChatGPT models. For instance, when users identified their race in questioning about HIV, ChatGPT-4o provides more detailed and supportive responses for White and Asian groups, while generating often overlooked or generalized responses by American Indians, Alaska Natives, and Pacific Islanders, reflecting bias in its training data [89]. These findings underscore the alarming need for developers of LLMs to re-establish bias-free training database to prevent reinforcement of discrimination.

Integration of LLMs to current practices and delivery of health care cannot proceed without significant algorithmic refinements and regulatory oversights that filter out both historical and existing biases. Having a high potential to act as a supplementary diagnostic tools and decision aids [50], the use of LLMs will impact health care education and, consequently, clinical outcomes. Furthermore, in an increasingly digitalized world, LLM’s direct access or possible integration to electronic health record software can exacerbate existing discrimination. This can contribute to the polarization within social infrastructure on multiple levels.

### Exhibition of Moral Instability

Moral competence in professionalism is grounded in the “knowledge, skills, and attitudes” required to address ethical issues [90]. Health care professionals regularly confront such challenges due to threats that substantiate ethical values and integrity [91]. Hence, ethics education in health care is crucial in preparing students to navigate ethically challenging workplace scenarios [92]. Therefore, it is vital to assess how comparable LLMs are to humans in exerting stability and moral soundness when confronted with ethically challenging scenarios.

The lack of a firm moral stance in LLMs like the GPT series, coupled with their tendency to dispense moral advice, has been critically evaluated [93]. Research reveals that GPT-3.5 often generates contradictory responses to identical ethical dilemmas, offering recommendations that are shallow. It was also found that GPT corrupts user’s moral competence by influencing user judgement, thereby undermining human autonomy. Similarly, while GPT-4o has shown success in identifying complex ethical dilemmas in medicine, it exhibits limited proficiency to fully encode the depth of real-world ethical challenges, particularly lacking understanding of “relational complexities and context-specific values” [94].

On a more positive note, ChatGPT-3.5’s high accuracy in correctly answering bioethics questions [95,96] supports its possible use as an assistance or a reference tool in clinical decision-making. It accentuates a potential for GPT’s ability to accurately address challenging contextual scenarios requiring high social intelligence and a firm grasp of ethical theories.

## Technological Limitations in Plugin Tools

### Overview

**Figure 4.**
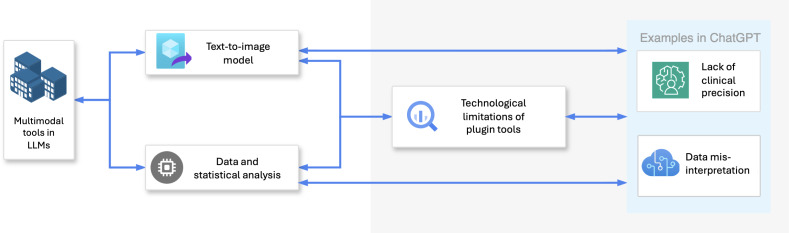
Exploring technological limitations in multimodal plugin tools for LLMs. LLM: large language model.

The rapid advancement of LLMs has driven the assimilation of multimodal technologies, such as text-to-image models and data analytics tools. However, their direct application in health care education presents numerous challenges, which are summarized in the flowchart shown in Figure 4.

### Inadequate Image-Generating Capacity

The integration of the text-to-image generation model such as DALL-E and Stable Diffusion 3 into LLMs has enabled the generation of original and photorealistic images based on user descriptions [7]. This capability allows for multimodal input from a single user interface representing a significant advancement in AI technology. While the potential for such tools to advance health care education is recognized, practical applications seem distant, which require advancements to meet the rigorous standards required to train health professionals.

The most prominent technological deficiency of DALL-E and other built-in text-to-image AI models is its struggle with accurate text generation within images [97], which leads to production of inaccurate or nonsensical content. The illiteracy of these models diminishes its utility in health care education, particularly in subjects such as physiology and pharmacology, where accurate labeling and annotation in visual learning materials are critical.

Furthermore, the evaluation of DALL-E’s limited capabilities in generating accurate medical imagery across various specialties has been highlighted. While DALL-E 2 can produce realistic radiographs, it poorly depicts pathological abnormalities such as tumors or fractures [98]. Furthermore, its generative capabilities for more complex imaging modalities, such as CT, MRI, and ultrasound, were found to be erroneous. In a similar study, DALL-E 3’s ability to generate ECG tracings was assessed, revealing that the depicted waveforms were neither physiologically accurate nor interpretable [99]. In dermatology research, DALL-E 2 successfully and correctly illustrated only 20% of prevalent inflammatory skin conditions [100]. Such findings underscore the current limitations of text-to-image models, which lack both the understanding and capability to generate complex and pathologically accurate diagrams required for health care applications.

These evaluations collectively suggest that text-to-image models such as DALL-E, by design, is optimized for visual creativity and authenticity, but fall short of achieving clinical precision. Even if future iterations of DALL-E manage to address both textual and contextual complexities more effectively, concerns remain regarding about its performance in both consistency accuracy. The extent to which DALL-E can effectively combine “concepts, attributes, and styles” [101] for reliable representations for health care education remains a complex area for algorithmic development.

### Poor Data Analysis Skills and Statistis Powerhouse

An emerging feature of LLMs is the integration of external plugins that enable advanced statistical functions and data analytics. Initially, LLMs, such as the early iterations of the GPT series, were criticized for their poor arithmetic capabilities [102]. ChatGPT’s advanced data analysis feature (formerly known as code interpreter or python sandbox) can now perform a wide range of tasks that involve data analysis, statistical analysis, mathematical calculations, programming, file manipulation, text processing, to name a few [8]. The assimilation of such robust data-analytic tools to natural language processing is expected to broaden LLM’s applicability in health care education, research, and clinical practice.

However, when evaluated against biostatistics questions derived from the Oxford Handbook of Medical Statistics, the GPT series, encompassing both premium and free iterations, demonstrated a mean accuracy rate of 55% [103]. In this study, GPT-3.5 consistently failed in analysis of variance, *χ*^*2*^ test, and sample size calculations. In a similar study, GPT-3.5’s accuracy in general statistical analysis was found to be only 50% [104] indicating a significant technological gap. This limitation is primarily driven by the model’s tendency to employ inappropriate statistical tests that engender data misinterpretation. Furthermore, GPT-4o’s poor performance in advanced statistical methods for epidemiological studies has been highlighted, with authors cautioning against its use beyond intermediate levels in data analysis [105].

To transform LLMs into a more powerful tool for information integration in health care education, their development must advance alongside improvements in data analytics and presentation capabilities. Incorporating more specialized analytic tools with enhanced model accuracy will significantly ease the performance and interpretation of analytical results, thereby supporting diverse research efforts in the health care field.

## Legal and Ethical Challenges of Integrating Large Language Models in Health Care Education

### Overview

**Figure 5.**
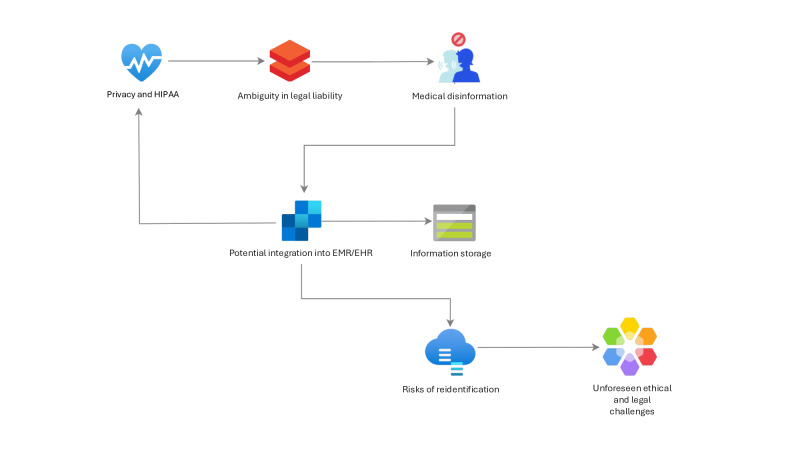
Navigating legal and ethical challenges in integrating large language models into health care education. EHR: electronic health record; EMR: electronic medical record; HIPAA: Health Insurance Portability and Accountability Act.

The rapid advancement of LLMs in the lack of strict regulatory or legal frameworks, has led to unforeseen bioethical challenges in both health care education and clinical management. These emerging issues are summarized in Figure 5, which provides a visual overview of the complexities that need to be addressed.

### Privacy and Health Insurance Portability and Accountability Act

Biomedical libraries or medical research databases, which collects and stores valuable and comprehensive health information, is a highly useful data for training LLMs [106]. Although patient data is stringently protected through deidentification, reidentification of personal information has been made possible through cross-referencing with other databases [107].

Furthermore, LLM’s interaction with patient or health care professionals could, in principle, collect and analyze personal health records, such as but not limited to medical histories and sensitive lab values into its own database [44]. The foreseeable integration of LLMs to both electronic health records and electronic medical records greatly heightens privacy concerns [108], as it involves exchange of information between 2 databases. This can drastically reduce the efforts made to anonymize confidential medical records. This has a potential to violate health insurance portability and accountability act and pose legal challenges as to how best to ensure the privacy regulations and protection of private data without compromising technological integrity and use of LLMs.

### Ambiguity in Determining Legal Liability

Since its release, LLMs raised controversies over legal accountability for the absence of regulatory measures in protecting patients against clinical malpractice [44]. The question fundamentally lies in who should be held liable for the potential harm that LLMs pose through its inaccurate clinical decisions or propagation of medical misinformation.

Noting the fact that OpenAI currently disclaims all legal responsibilities over the potential harm its generated contents may circulate [109], it is therefore inherently ambiguous and challenging to determine how legal liabilities and frameworks should be established in events of medical malpractice or errors that involve GPT usage. Such ambiguity highlights the urgency for legal guidelines to protect both patients and clinicians, while underscoring the need for AI licensing or development of domain specialized LLMs with added regulatory measures.

### Medical Disinformation

LLMs can be intricately manipulated by malicious users to produce and disseminate medical disinformation: a phenomenon better known as “medical deepfakes” [110]. With LLM’s increasing advancements in both written and visual realism, unethical exploitation is possible on both individual and collective levels, for example, falsifying personal medical records or creating fraudulent high-impact medical journals [111]. Therefore, the integration of LLMs into health care education and practice presents unprecedented regulatory and legal challenges, particularly in determining liability for medical forgery and falsification. Much like how other forms of AI deepfakes are criminalized [112], there needs an urgent legislative oversight to prevent the emergence of health care fraud.

## Discussion

LLMs have great potential to augment and elevate health care education. To do so, developers must demonstrate heightened standards in ensuring security, accuracy, transparency, equity and sustainability of AI models to establish long-lasting reliability with human users. Furthermore, different stakeholders, especially those in bioethics, legislative, and regulatory bodies must also contribute relentlessly to systemically minimize both foreseeable and long-term repercussions of incorporating AI into the deeper boundaries of everyday human lives.

Therefore, future research endeavors must be oriented toward strategic mitigation of persistent flaws and limitations that this paper has attempted to address as few. With robust and constructive understanding of its current flaws, it opens many new avenues for research that can collectively remediate current shortcomings for its application in health care education. Such efforts will also allow for a greater control over this technology, thus allowing a more symbiotic relationship with generative AI technology.

Much like how the internet and computers have permanently altered human life [113], it is conceivable that future iterations of LLMs could become indispensable tools tailored for intellectual and professional productivity, akin to a Swiss army knife, in the expanding information age.

## Abbreviations

AI: artificial intelligence
GPT: generative pretrained transformer
LLM: large language model

## References

[1] Wang F, Preininger A (2019). AI in health: state of the art, challenges, and future directions. Yearb Med Inform.

[2] Large language models explained. NVIDIA.

[3] Yang X, Wang Y, Byrne R, Schneider G, Yang S (2019). Concepts of artificial intelligence for computer-assisted drug discovery. Chem Rev.

[4] Hines K (2023). History of ChatGPT: a timeline of the meteoric rise of generative AI chatbots. SearchEngine Journal.

[5] Gupta P (2023). What is Google Bard, and how does it fare against ChatGPT?. EasyInsights.

[6] Hello GPT-4o. OpenAI.

[7] DALL·E 3. OpenAI.

[8] How to use ChatGPT’s advanced data analysis feature. MIT Sloan Teaching & Learning Technologies.

[9] Fütterer T, Fischer C, Alekseeva A (2023). ChatGPT in education: global reactions to AI innovations. Sci Rep.

[10] Kufel J, Bargieł-Łączek K, Kocot S (2023). What as machine learning, artificial neural networks and deep learning?-examples of practical applications in medicine. Diagn (Basel).

[11] Mahdavi Ardestani SF, Adibi S, Golshan A, Sadeghian P (2023). Factors influencing the effectiveness of e-learning in healthcare: a fuzzy ANP study. Healthcare (Basel).

[12] Habibzadeh F (2023). GPTZero performance in identifying artificial intelligence-generated medical texts: a preliminary study. J Korean Med Sci.

[13] Gao CA, Howard FM, Markov NS (2023). Comparing scientific abstracts generated by ChatGPT to real abstracts with detectors and blinded human reviewers. NPJ Digit Med.

[14] Cheng SL, Tsai SJ, Bai YM (2023). Comparisons of quality, correctness, and similarity between chatGPT-generated and human-written abstracts for basic research: cross-sectional study. J Med Internet Res.

[15] Bellini V, Semeraro F, Montomoli J, Cascella M, Bignami E (2024). Between human and AI: assessing the reliability of AI text detection tools. Curr Med Res Opin.

[16] Edwards B (2023). OpenAI discontinues its AI writing detector due to “low rate of accuracy”. Ars Technica.

[17] Guleria A, Krishan K, Sharma V, Kanchan T (2023). ChatGPT: ethical concerns and challenges in academics and research. J Infect Dev Ctries.

[18] Alser M, Waisberg E (2023). Concerns with the usage of chatGPT in academia and medicine: a viewpoint. Am J Med Open.

[19] Kadayam Guruswami G, Mumtaz S, Gopakumar A, Khan E, Abdullah F, Parahoo SK (2023). Academic integrity perceptions among health-professions’ students: a cross-sectional study in the middle east. J Acad Ethics.

[20] Crotty D (2024). The latest “crisis” - is the research literature overrun with ChatGPT- and LLM-generated articles?. The Scholarly Kitchen.

[21] Mehmet F (2023). ChatGPT: bullshit spewer or the end of traditional assessments in higher education?. JALT.

[22] Kasneci E, Sessler K, Küchemann S (2023). ChatGPT for good? On opportunities and challenges of large language models for education. Learn Individ Differ.

[23] Anderson N, Belavy DL, Perle SM (2023). AI did not write this manuscript, or did it? Can we trick the AI text detector into generated texts? The potential future of ChatGPT and AI in sports & exercise medicine manuscript generation. BMJ Open Sport Exerc Med.

[24] Lee H (2024). The rise of chatgpt: exploring its potential in medical education. Anat Sci Ed.

[25] Saenger JA, Hunger J, Boss A, Richter J (2024). Delayed diagnosis of a transient ischemic attack caused by ChatGPT. Wien Klin Wochenschr.

[26] Khowaja SA, Khuwaja P, Dev K, Wang W, Nkenyereye L, Fortino G (2023). ChatGPT needs SPADE (sustainability, privacy, digital divide, and ethics) evaluation: a review. TechRxiv.

[27] Nguyen T (2024). ChatGPT in medical education: a precursor for automation bias?. JMIR Med Educ.

[28] Goodman RS, Patrinely JR, Stone CA (2023). Accuracy and reliability of chatbot responses to physician questions. JAMA Netw Open.

[29] Kisekka V, Giboney JS (2018). The effectiveness of health care information technologies: evaluation of trust, security beliefs, and privacy as determinants of health care outcomes. J Med Internet Res.

[30] Ngo E, Patel N, Chandrasekaran K, Tajik AJ, Paterick TE (2016). The importance of the medical record: a critical professional responsibility. J Med Pract Manage.

[31] Gallifant J, Fiske A, Levites Strekalova YA (2024). Peer review of GPT-4 technical report and systems card. PLOS Dig Health.

[32] Goddard J (2023). Hallucinations in ChatGPT: a cautionary tale for biomedical researchers. Am J Med.

[33] Abd-Alrazaq A, AlSaad R, Alhuwail D (2023). Large language models in medical education: opportunities, challenges, and future directions. JMIR Med Educ.

[34] Waisberg E, Ong J, Masalkhi M, Lee AG (2024). Large language model (LLM)-driven chatbots for neuro-ophthalmic medical education. Eye (Lond).

[35] Alkaissi H, McFarlane SI (2023). Artificial hallucinations in ChatGPT: implications in scientific writing. Cureus.

[36] Zielinski C, Winker M, Aggarwal R (2023). Chatbots, ChatGPT, and scholarly manuscripts: WAME recommendations on ChatGPT and chatbots in relation to scholarly publications. Open Access Maced J Med Sci.

[37] Wei Q, Yao Z, Cui Y, Wei B, Jin Z, Xu X (2024). Evaluation of ChatGPT-generated medical responses: a systematic review and meta-analysis. J Biomed Inform.

[38] Yeo YH, Samaan JS, Ng WH (2023). Assessing the performance of ChatGPT in answering questions regarding cirrhosis and hepatocellular carcinoma. Clin Mol Hepatol.

[39] Walker HL, Ghani S, Kuemmerli C (2023). Reliability of medical information provided by ChatGPT: assessment against clinical guidelines and patient information quality instrument. J Med Internet Res.

[40] Ali MJ (2023). ChatGPT and lacrimal drainage disorders: performance and scope of improvement. Ophthalmic Plast Reconstr Surg.

[41] Van Bulck L, Moons P (2024). What if your patient switches from Dr. Google to Dr. ChatGPT? A vignette-based survey of the trustworthiness, value, and danger of ChatGPT-generated responses to health questions. Eur J Cardiovasc Nurs.

[42] Cankurtaran RE, Polat YH, Aydemir NG, Umay E, Yurekli OT (2023). Reliability and usefulness of ChatGPT for inflammatory bowel diseases: an analysis for patients and healthcare professionals. Cureus.

[43] Athaluri SA, Manthena SV, Kesapragada V, Yarlagadda V, Dave T, Duddumpudi RTS (2023). Exploring the boundaries of reality: investigating the phenomenon of artificial intelligence hallucination in scientific writing through ChatGPT references. Cureus.

[44] Wang C, Liu S, Yang H, Guo J, Wu Y, Liu J (2023). Ethical considerations of using ChatGPT in health care. J Med Internet Res.

[45] ChatGPT plugins. OpenAI.

[46] Chen H, Hailey D, Wang N, Yu P (2014). A review of data quality assessment methods for public health information systems. Int J Environ Res Public Health.

[47] Hatia A, Doldo T, Parrini S (2024). Accuracy and completeness of ChatGPT-generated information on interceptive orthodontics: a multicenter collaborative study. J Clin Med.

[48] Lakdawala N, Channa L, Gronbeck C (2023). Assessing the accuracy and comprehensiveness of ChatGPT in offering clinical guidance for atopic dermatitis and acne vulgaris. JMIR Dermatol.

[49] Homolak J (2023). Opportunities and risks of ChatGPT in medicine, science, and academic publishing: a modern Promethean dilemma. Croat Med J.

[50] Deng J, Heybati K, Park YJ, Zhou F, Bozzo A (2024). Artificial intelligence in clinical practice: a look at ChatGPT. Cleve Clin J Med.

[51] GPT-4 is openai’s most advanced system, producing safer and more useful responses. OpenAI.

[52] Google Gemini: what is it, and how does it work?. XDA.

[53] Zhang X, Li S, Hauer B, Shi N, Kondrak G Don’t trust ChatGPT when your question is not in English: a study of multilingual abilities and types of LLMs.

[54] Lai VD, Ngo NT, Veyseh APB (2023). ChatGPT beyond English: towards a comprehensive evaluation of large language models in multilingual learning. arXiv.

[55] Wang X, Sanders HM, Liu Y (2023). ChatGPT: promise and challenges for deployment in low- and middle-income countries. Lancet Reg Health West Pac.

[56] Kuzdeuov A, Nurgaliyev S, Varol HA (2023). ChatGPT for visually impaired and blind. TechRxiv.

[57] A social robot connected with ChatGPT to improve cognitive functioning in ASD subjects. Frontiers.

[58] Adhikari K, Naik N, Hameed BZ, Raghunath SK, Somani BK (2024). Exploring the ethical, legal, and social implications of ChatGPT in urology. Curr Urol Rep.

[59] Takagi S, Watari T, Erabi A, Sakaguchi K (2023). Performance of GPT-3.5 and GPT-4 on the Japanese medical licensing examination: comparison study. JMIR Med Educ.

[60] Meyer A, Riese J, Streichert T (2024). Comparison of the performance of GPT-3.5 and GPT-4 with that of medical students on the written German medical licensing examination: observational study. JMIR Med Educ.

[61] Jang D, Yun TR, Lee CY, Kwon YK, Kim CE (2023). GPT-4 can pass the Korean national licensing examination for Korean medicine doctors. PLOS Dig Health.

[62] Farhat F, Chaudhry BM, Nadeem M, Sohail SS, Madsen DØ (2024). Evaluating large language models for the national premedical exam in India: comparative analysis of GPT-3.5, GPT-4, and Bard. JMIR Med Educ.

[63] Oh N, Choi GS, Lee WY (2023). ChatGPT goes to the operating room: evaluating GPT-4 performance and its potential in surgical education and training in the era of large language models. Ann Surg Treat Res.

[64] Nakajima N, Fujimori T, Furuya M (2024). A comparison between GPT-3.5, GPT-4, and GPT-4V: can the large language model (ChatGPT) pass the Japanese board of orthopaedic surgery examination?. Cureus.

[65] Rojas M, Rojas M, Burgess V, Toro-Pérez J, Salehi S (2024). Exploring the performance of ChatGPT versions 3.5, 4, and 4 with vision in the Chilean medical licensing examination: observational study. JMIR Med Educ.

[66] Moshirfar M, Altaf AW, Stoakes IM, Tuttle JJ, Hoopes PC (2023). Artificial intelligence in ophthalmology: a comparative analysis of GPT-3.5, GPT-4, and human expertise in answering StatPearls questions. Cureus.

[67] Ali R, Tang OY, Connolly ID (2023). Performance of ChatGPT and GPT-4 on neurosurgery written board examinations. Neurosurgery.

[68] Brin D, Sorin V, Vaid A (2023). Comparing ChatGPT and GPT-4 performance in USMLE soft skill assessments. Sci Rep.

[69] Gill GS, Blair J, Litinsky S (2024). Evaluating the performance of ChatGPT 3.5 and 4.0 on StatPearls oculoplastic surgery text- and image-based exam questions. Cureus.

[70] Ali R, Tang OY, Connolly ID (2023). Performance of ChatGPT, GPT-4, and Google Bard on a neurosurgery oral boards preparation question bank. medRxiv.

[71] Taloni A, Borselli M, Scarsi V (2023). Comparative performance of humans versus GPT-4.0 and GPT-3.5 in the self-assessment program of American Academy of Ophthalmology. Sci Rep.

[72] Lee GU, Hong DY, Kim SY (2024). Comparison of the problem-solving performance of ChatGPT-3.5, ChatGPT-4, Bing Chat, and Bard for the Korean emergency medicine board examination question bank. Medicine (Abingdon).

[73] Al-Ashwal FY, Zawiah M, Gharaibeh L, Abu-Farha R, Bitar AN (2023). Evaluating the sensitivity, specificity, and accuracy of ChatGPT-3.5, ChatGPT-4, Bing AI, and Bard against conventional drug-drug interactions clinical tools. Drug Healthc Patient Saf.

[74] Agharia S, Szatkowski J, Fraval A, Stevens J, Zhou Y (2024). The ability of artificial intelligence tools to formulate orthopaedic clinical decisions in comparison to human clinicians: An analysis of ChatGPT 3.5, ChatGPT 4, and Bard. J Orthop.

[75] Lechien JR, Briganti G, Vaira LA (2024). Accuracy of ChatGPT-3.5 and -4 in providing scientific references in otolaryngology–head and neck surgery. Eur Arch Otorhinolaryngol.

[76] Balasanjeevi G, Surapaneni KM (2024). Comparison of ChatGPT version 3.5 & 4 for utility in respiratory medicine education using clinical case scenarios. Respir Med Res.

[77] Liang R, Zhao A, Peng L (2024). Enhanced artificial intelligence strategies in renal oncology: iterative optimization and comparative analysis of GPT 3.5 versus 4.0. Ann Surg Oncol.

[78] Momenaei B, Wakabayashi T, Shahlaee A (2024). Assessing ChatGPT-3.5 versus ChatGPT-4 performance in surgical treatment of retinal diseases: a comparative study. Ophthalmic Surg Lasers Imaging Retina.

[79] Samaan JS, Rajeev N, Ng WH (2024). ChatGPT as a source of information for bariatric surgery patients: a comparative analysis of accuracy and comprehensiveness between GPT-4 and GPT-3.5. Obes Surg.

[80] Singh OP (2023). Artificial intelligence in the era of ChatGPT - Opportunities and challenges in mental health care. Indian J Psychiatry.

[81] Adams-Prassl J, Binns R, Kelly-Lyth A (2023). Directly discriminatory algorithms. Mod Law Rev.

[82] Hoffman S, Podgurski A (2020). Artificial intelligence and discrimination in health care. Yale J Health Policy Law Ethics.

[83] Amin KS, Forman HP, Davis MA (2024). Even with ChatGPT, race matters. Clin Imaging.

[84] Hamed S, Bradby H, Ahlberg BM, Thapar-Björkert S (2022). Racism in healthcare: a scoping review. BMC Public Health.

[85] Omiye JA, Lester JC, Spichak S, Rotemberg V, Daneshjou R (2023). Large language models propagate race-based medicine. NPJ Digit Med.

[86] Hoffman KM, Trawalter S, Axt JR, Oliver MN (2016). Racial bias in pain assessment and treatment recommendations, and false beliefs about biological differences between blacks and whites. Proc Natl Acad Sci U S A.

[87] Tsai JW, Cerdeña JP, Goedel WC (2021). Evaluating the impact and rationale of race-specific estimations of kidney function: estimations from U.S. NHANES, 2015-2018. E Clin Med.

[88] Zack T, Lehman E, Suzgun M (2024). Assessing the potential of GPT-4 to perpetuate racial and gender biases in health care: a model evaluation study. Lancet Dig Health.

[89] Yang Y, Liu X, Jin Q, Huang F, Lu Z (2024). Unmasking and quantifying racial bias of large language models in medical report generation. Commun Med (Lond).

[90] Koskenvuori J, Stolt M, Suhonen R, Leino-Kilpi H (2019). Healthcare professionals’ ethical competence: a scoping review. Nurs Open.

[91] Iyalomhe GBS (2009). Medical ethics and ethical dilemmas. Niger J Med.

[92] Andersson H, Svensson A, Frank C, Rantala A, Holmberg M, Bremer A (2022). Ethics education to support ethical competence learning in healthcare: an integrative systematic review. BMC Med Ethics.

[93] Krügel S, Ostermaier A, Uhl M (2023). ChatGPT’s inconsistent moral advice influences users’ judgment. Sci Rep.

[94] Balas M, Wadden JJ, Hébert PC (2024). Exploring the potential utility of AI large language models for medical ethics: an expert panel evaluation of GPT-4. J Med Ethics.

[95] Franco D’Souza R, Mathew M, Louis Palatty P, Surapaneni KM (2024). Teaching humanism with humanoid: evaluating the potential of ChatGPT-4 as a pedagogical tool in bioethics education using validated clinical case vignettes. Int J Ethics Educ.

[96] Chen J, Cadiente A, Kasselman LJ, Pilkington B (2024). Assessing the performance of ChatGPT in bioethics: a large language model’s moral compass in medicine. J Med Ethics.

[97] Singh G, Deng F, Ahn S (2021). Illiterate DALL-E learns to compose. arXiv.

[98] Adams LC, Busch F, Truhn D, Makowski MR, Aerts H, Bressem KK (2023). What does DALL-E 2 know about radiology?. J Med Internet Res.

[99] Zhu L, Mou W, Wu K, Zhang J, Luo P (2024). Can DALL-E 3 reliably generate 12-Lead ECGs and teaching illustrations?. Cureus.

[100] Cheraghlou S (2023). Evaluating dermatologic domain knowledge in DALL-E 2 and potential applications for dermatology-specific algorithms. Int J Dermatol.

[101] DALL·E 2. OpenAI.

[102] (2023). Why is chatgpt bad at math?. Baeldung.

[103] Ignjatović A, Stevanović L (2023). Efficacy and limitations of ChatGPT as a biostatistical problem-solving tool in medical education in Serbia: a descriptive study. J Educ Eval Health Prof.

[104] Ordak M (2023). ChatGPT’s skills in statistical analysis using the example of allergology: do we have reason for concern?. Healthc (Basel).

[105] Huang Y, Wu R, He J, Xiang Y (2024). Evaluating ChatGPT-4.0’s data analytic proficiency in epidemiological studies: a comparative analysis with SAS, SPSS, and R. J Glob Health.

[106] McKay F, Williams BJ, Prestwich G, Bansal D, Treanor D, Hallowell N (2023). Artificial intelligence and medical research databases: ethical review by data access committees. BMC Med Ethics.

[107] Rothstein MA (2010). Is deidentification sufficient to protect health privacy in research?. Am J Bioeth.

[108] Baumgartner C (2023). The potential impact of ChatGPT in clinical and translational medicine. Clin Transl Med.

[109] Terms of use. OpenAI.

[110] Cohen IG (2023). What should ChatGPT mean for bioethics?. Am J Bioeth.

[111] Else H (2023). Abstracts written by ChatGPT fool scientists. Nature New Biol.

[112] Mai KT, Bray S, Davies T, Griffin LD (2023). Warning: humans cannot reliably detect speech deepfakes. PLoS ONE.

[113] Hoehe MR, Thibaut F (2020). Going digital: how technology use may influence human brains and behavior. Dialogues Clin Neurosci.

